# Interaction between climatic, environmental, and demographic factors on cholera outbreaks in Kenya

**DOI:** 10.1186/2049-9957-3-37

**Published:** 2014-10-01

**Authors:** James D Stoltzfus, Jane Y Carter, Muge Akpinar-Elci, Martin Matu, Victoria Kimotho, Mark J Giganti, Daniel Langat, Omur Cinar Elci

**Affiliations:** School of Medicine, Department of Public Health and Preventive Medicine, St. George’s University (SGU), West Indies, Grenada; African Medical and Research Foundation (AMREF), Nairobi, Kenya; Center for Global Health, College of Health Sciences, Old Dominion University, Norfolk, VA USA

**Keywords:** Cholera, Education, Health infrastructure, Population density, Poverty, Rainfall, Waste disposal, Water supply

## Abstract

**Background:**

Cholera remains an important public health concern in developing countries including Kenya where 11,769 cases and 274 deaths were reported in 2009 according to the World Health Organization (WHO). This ecological study investigates the impact of various climatic, environmental, and demographic variables on the spatial distribution of cholera cases in Kenya.

**Methods:**

District-level data was gathered from Kenya’s Division of Disease Surveillance and Response, the Meteorological Department, and the National Bureau of Statistics. The data included the entire population of Kenya from 1999 to 2009.

**Results:**

Multivariate analyses showed that districts had an increased risk of cholera outbreaks when a greater proportion of the population lived more than five kilometers from a health facility (RR: 1.025 per 1% increase; 95% CI: 1.010, 1.039), bordered a body of water (RR: 5.5; 95% CI: 2.472, 12.404), experienced increased rainfall from October to December (RR: 1.003 per 1 mm increase; 95% CI: 1.001, 1.005), and experienced decreased rainfall from April to June (RR: 0.996 per 1 mm increase; 95% CI: 0.992, 0.999). There was no detectable association between cholera and population density, poverty, availability of piped water, waste disposal methods, rainfall from January to March, or rainfall from July to September.

**Conclusion:**

Bordering a large body of water, lack of health facilities nearby, and changes in rainfall were significantly associated with an increased risk of cholera in Kenya.

**Electronic supplementary material:**

The online version of this article (doi:10.1186/2049-9957-3-37) contains supplementary material, which is available to authorized users.

## Multilingual abstracts

Please see Additional file 1 for translations of the abstract into the six official working languages of the United Nations.

## Background

Cholera continues to be a global public health threat and one of the key indicators of poor social development [1–3]. The worldwide incidence of cholera has risen in the last decade with 589,854 cases and 7,816 deaths reported in 2011, an increase of 220.0% cases and 186.5% deaths from 2001 [1–3]. Since 2007, Kenya has experienced an increase in cholera outbreaks [4, 5]. Cholera cases from Africa represented one third of the global total in 2011 [6]. The most commonly cited risk factors for cholera outbreaks worldwide were water source contamination, rainfall and flooding, and refugee settings, mainly in the context of complex emergency situations. In East Africa, refugee settings and water source contamination were most commonly identified [1–3, 7].

Climatic factors such as rainfall, water and sea surface temperatures, and the El Niño Southern Oscillation events have a significant impact on the incidence of cholera [8–10]. In East Africa, cholera epidemics have been closely associated with the El Niño years [11–15], and were aggravated by the low socioeconomic status, inadequate healthcare systems [5, 14], drinking water from lakes [15, 16], and inadequate sanitary facilities [15]. Some studies have shown that cholera cases occur in highly urbanized, densely populated areas close to water bodies [17–19], while others have shown a higher incidence in small, rural populations with high poverty levels, low urbanization, and lack of sanitation [20–22]. Both populations had low educational levels and environmental factors, particularly water supply and the method of human waste disposal, play a significant role in cholera outbreaks [23–25]. However, there is little data on how different variables interact and contribute to cholera outbreaks in Kenya. This ecological study aims to identify the demographic, climatic, and environmental factors that make Kenya especially vulnerable to cholera outbreaks. This study investigates the role of population density, education levels, poverty, religion, water supply, human waste disposal, distance to the nearest health facility, geographical location near large bodies of water, and rainfall on the incidence and spatial distribution of cholera cases in Kenya over an 11-year time period (1999–2009).

## Methods

### Data collection

The study reviewed selected climatic, environmental, and demographic factors that may be associated with cholera outbreaks in Kenya. The data covered all eight provinces and 69 administrative districts in the country, and included the entire population of Kenya. Data on cholera cases by year and district from 1999 to 2009 were collected from the Division of Disease Surveillance and Response (DDSR), Ministry of Public Health and Sanitation, Kenya. Data from 1999 and 2000 were approximate figures reported in intervals of 50; data was not available for the years 2002, 2004, and 2006. For 1999, 2005, and 2009, dates of cholera outbreaks were also obtained.

A cholera case was defined as either: (i) profuse effortless watery diarrhea (more than three watery stools in 24 hours) of sudden onset with or without vomiting in a person over five years of age, or (ii) all cases with acute watery diarrhea in a person over two years of age residing in an area experiencing an outbreak. Not all reported cases of cholera were confirmed by microbiological examination.

Demographic and environmental characteristics for each district were accessed from the 2005/2006 Integrated Household Budget Survey conducted by the Kenya National Bureau of Statistics. The demographic data included district-level populations, population density, the percentage of the population older than six years of age who never attended school, the percentage of the population practicing various religions, and the percentage of the population falling below the rural poverty line (under $13.34 USD/per day). The environmental data included the percentage of the population without a piped water supply, the percentage of the population with unsafe sanitary facilities (defined as anything other than flush toilet, ventilated improved pit latrine, or pit latrine), and the percentage of the population living five kilometres (km) or more from a health facility. Monthly rainfall amounts and mean high temperatures for each district from 1999 to 2009 were provided by the Kenya Meteorological Department in Nairobi, Kenya. Maps portraying the water bodies and floodplains in Kenya were obtained from the World Resource Institute. Fresh water resources and body of waters used as a water resource were identified. The datasets were created by the Food and Agriculture Organization (FAO) from FAO’s Africover Multipurpose Land Cover Databases for Kenya (2000).

This study received funding support from St. George’s University, and the study methodology was reviewed and approved by the Ethical and Scientific Review Committee (ESRC) of the African Medical and Research Foundation and the Institutional Review Board (IRB) of St. George’s University.

### Data analysis

We defined the following district-level characteristics as possible risk factors for cholera outbreaks: population density; percentage of the population who never attended school, fall below the rural poverty line, live without piped water, live with unsafe sanitary facilities, live five km or more from a health facility, practice various religions; location bordering a body of water; quarterly mean high temperature; and quarterly total rainfall. Our primary outcome of interest was the incidence of cholera.

Geographic Information System (GIS)-based spatial analysis depicting the spatial distribution of cholera cases were generated in ArcView 3.2 (ESRI, Redlands, CA, USA). Additional maps were also generated to illustrate the spatial distribution of each demographic and environmental variable being investigated.

The districts were divided into two groups based on the occurrence of cholera cases during the study period and tested for differences in demographic and environmental characteristics using Wilcoxon Rank-Sum tests for categorical-continuous variables and Chi-Square tests for continuous-categorical variables. Because of variations in the number of at-risk persons among districts, we standardized the number of cholera cases per 100,000 persons per year per district. A repeated measures analysis, which accounts for clustering by district, was performed using Generalized Estimating Equations with a log link function and an exchangeable correlation structure. Due to concerns about multicolinearity, we simultaneously calculated variance inflation factors (VIFs) for all covariates of interest; any variables with a VIF greater than five were excluded from the final model. Due to unavailability of temperature data for 43 out of the 69 districts (62.3%), we also excluded the covariates representing quarterly temperature. We report relative risks and corresponding 95% confidence intervals (CIs). All statistical analyses were performed using R software version 2.12.1 (http://www.r-project.org).

## Results

**Figure 1:**
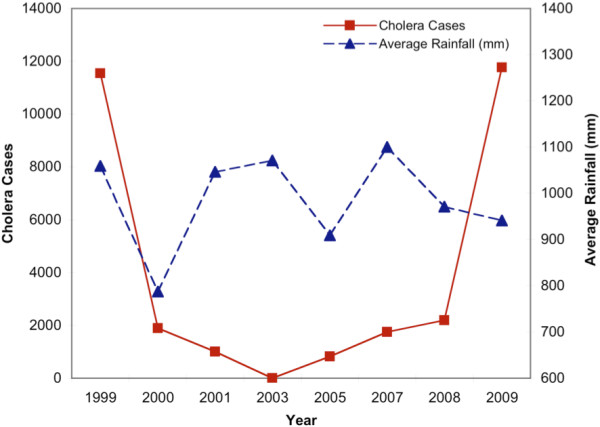
**Reported number of cholera cases and average rainfall across the 69 districts in Kenya, by year.**

**Figure 2:**
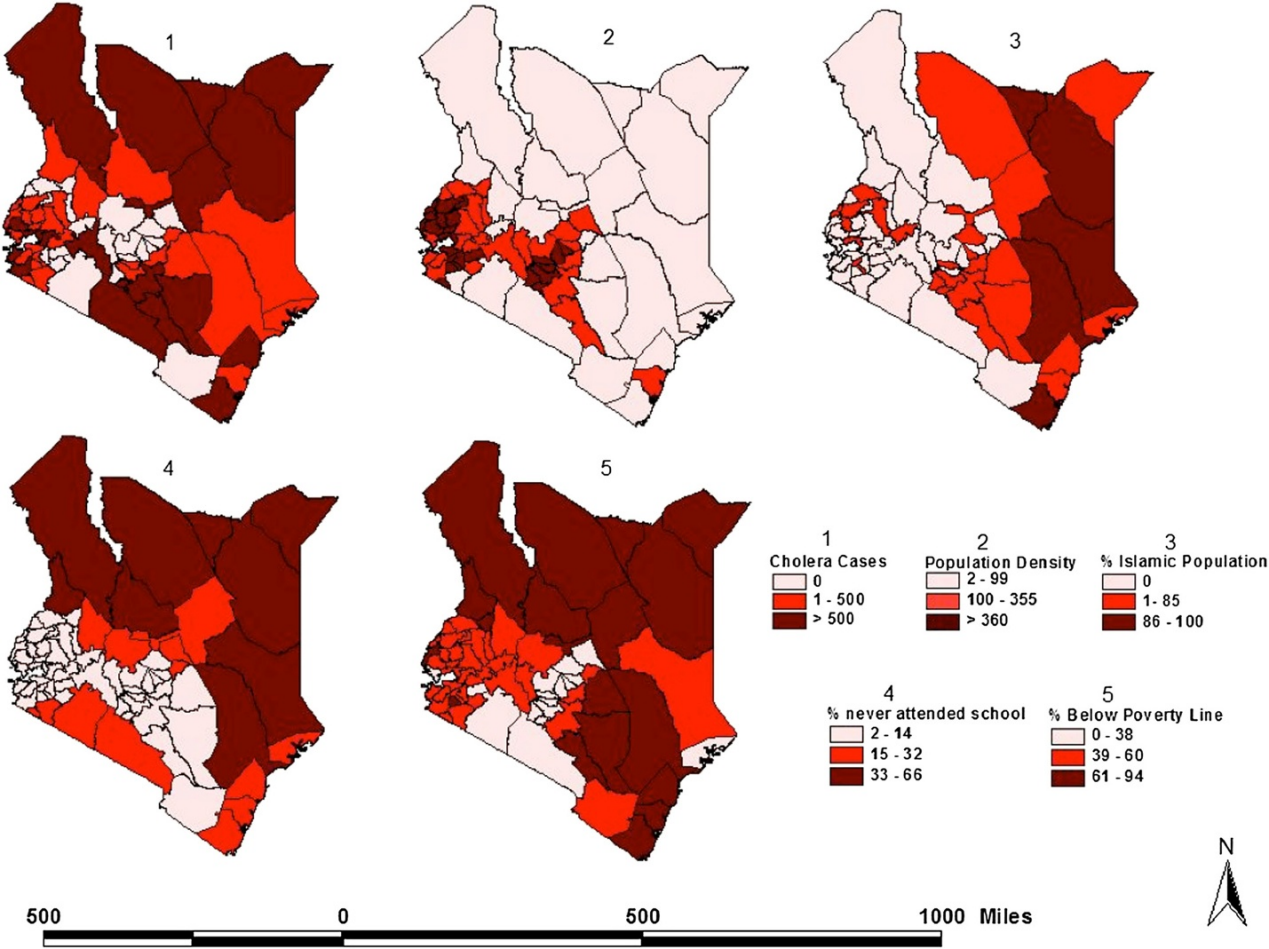
**District-level distribution of cholera cases and various demographic characteristics in Kenya.** **(1)** Combined number of cholera cases reported per district in eight separate years: 1999–2001, 2003, 2005, 2007–2009. **(2)** Population density (persons/km^2^), per district. **(3)** Percentage of the Muslim population, per district. **(4)** Percentage of the population older than six years of age who never attended school, per district. **(5)** Percentage of the population per district below the rural poverty line (set at $1,239 Ksh [$13.34 USD] per day). *Sources:* Cholera data: Division of Disease Surveillance and Response. All other variables: 2005/06 Integrated Household Budget Survey.

**Figure 3:**
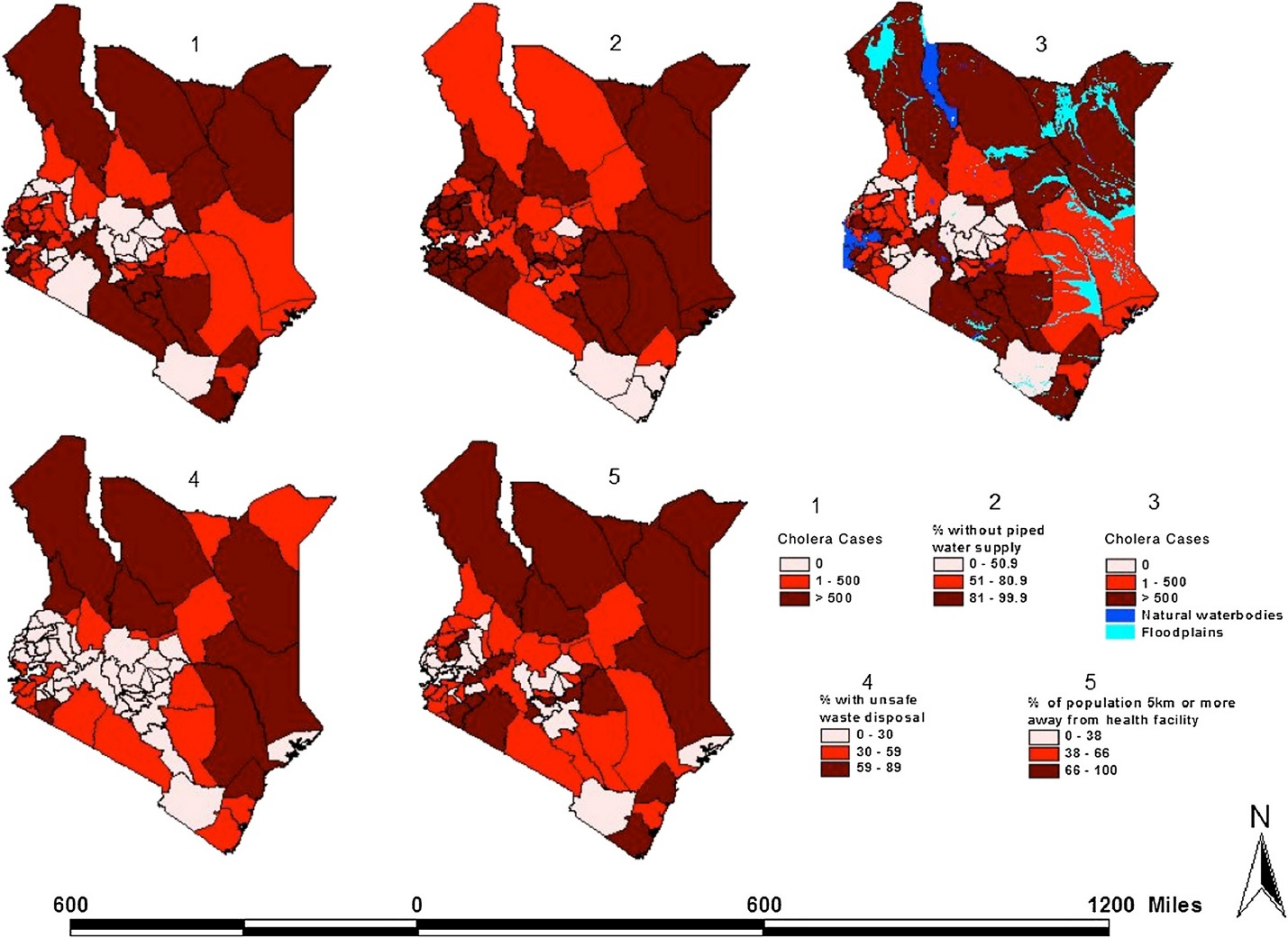
**District-level distribution of cholera cases and various environmental characteristics in Kenya.** **(1)** Combined number of cholera cases reported per district in eight separate years: 1999–2001, 2003, 2005, 2007–2009. **(2)** Percentage of the population per district without piped water supply. **(3)** Same as Map 1, plus the distribution of water bodies and flood plains. **(4)** Percentage of the population per district with unsafe sanitary facilities defined as anything other than flush toilet, ventilated improved pit latrine, or pit latrine. (5) Percentage of the population per district who lives five or more km away from a health facility. *Sources:* Cholera data: Division of Disease Surveillance and Response. Water bodies and floodplain data: World Resource Institute. All other variables: 2005/06 Integrated Household Budget Survey.

According to national data for the years 1999 to 2009 (excluding 2002, 2004, and 2006), 31,001 cases of cholera were reported among the 35,514,544 at-risk persons living in 69 districts in Kenya. The annual incidence was 10.91 cholera cases per 100,000 persons [95% (CI): 10.79, 11.03]. Cholera incidence during 12-month intervals varied across the study period, ranging from zero reported cases in 2003 to 11,769 cases in 2009 (see Figure 1). The mean yearly rainfall from the available data for 465 out of the 552 districts/years (84.2%) was 986.2 mm [minimum: 787.4 mm in 2000; maximum: 1100 mm in 2007] (see Figure 1). The spatial variation in cholera incidence, demographic characteristics, and environmental characteristics in all 69 districts is mapped in Figures 2 and 3.

Forty-eight out of 69 (69.6%) Kenyan districts reported at least one cholera case during the study period. Compared to districts with no reported cholera cases, districts with at least one reported case were more likely to have higher median levels of no schooling (11.5% vs. 8.0%; p = 0.011), poverty (51.8% vs. 45.3% p = 0.003), and unsafe sanitary facilities (32.5% vs. 3%; p = 0.001), and higher median percentages of the population who were predominantly Muslim (0% vs. 0.6%; p = 0.044) or did not live within five km of a health facility (61.4% vs. 43.5%; p = 0.013). While 62.5% of districts affected by cholera did not border a large body of water, 37.5% of districts affected by cholera bordered the Indian Ocean (12.5%), Lake Victoria, or Lake Turkana (25%), and this observed difference was statistically significant (p = 0.003). District-level characteristics are shown in Table 1 and their spatial distribution is shown in Figures 2 and 3.

**Table 1:** **Demographic and ecological characteristics of the 69 districts in Kenya**

	Districts with no documented cholera cases (n = 21)	Districts with documented cholera cases (n = 48)	P-value
*Population density (median, IQR)*	229.7 (134.7 – 395.5)	187.4 (36.2 – 430)	0.403*
			0.299**
0–50	3 (14.3%)	16 (33.3%)	
51–200	5 (23.8%)	9 (18.8%)	
201–500	10 (47.6%)	14 (29.2%)	
501–1000	3 (14.3%)	6 (12.5%)	
1000+	0 (0%)	3 (6.3%)	
*No schooling,% (median, IQR)*	8 (6.7 – 11.8)	11.5 (8.7 – 25.1)	0.011*
			0.052**
0–15%	18 (85.7%)	29 (60.4%)	
16–30%	3 (14.3%)	9 (18.8%)	
31–100%	0 (0%)	10 (20.8%)	
*Poverty,% (median, IQR)*	45.3 (30.8 – 50.3)	51.8 (42.7 – 68.7)	0.003*
			0.003**
0–33%	9 (42.9%)	5 (10.4%)	
34–66%	11 (52.4%)	30 (62.5%)	
67–100%	1 (4.8%)	13 (27.1%)	
*Unsafe sanitary facilities,% (median, IQR)*	3 (0.5 – 12)	32.5 (5.4 – 52.8)	0.001*
			0.016**
0–20%	18 (85.7%)	22 (45.8%)	
21–40%	2 (9.5%)	9 (18.8%)	
41–60%	1 (4.8%)	8 (16.7%)	
61–100%	0 (0%)	9 (18.8%)	
*No piped water,% (median, IQR)*	71.9 (59.9 – 84)	86 (72.9 – 93.8)	0.083*
			0.038**
0–50%	2 (9.5%)	4 (8.3%)	
51–75%	10 (47.6%)	9 (18.8%)	
76–100%	9 (42.9%)	35 (72.9%)	
*No health facility within 5 km,% (median, IQR)*	43.5 (32.2 – 51.8)	61.4 (45.5 – 78)	0.013*
			0.07**
0–25%	3 (14.3%)	6 (12.5%)	
26–50%	11 (52.4%)	11 (22.9%)	
51–75%	5 (23.8%)	17 (35.4%)	
76–100%	2 (9.5%)	14 (29.2%)	
*Muslim% (median, IQR)*	0 (0 – 0.9)	0.6 (0 – 15.1)	0.044*
			0.107**
0%	15 (71.4%)	23 (47.9%)	
1–10%	5 (23.8%)	11 (22.9%)	
11–20%	1 (4.8%)	4 (8.3%)	
21–100%	0 (0%)	10 (20.8%)	
*Large body of water*			0.003**
No	21 (100%)	30 (62.5%)	
Ocean	0 (0%)	6 (12.5%)	
Fresh	0 (0%)	12(25%)	

Bolded values represents significant statistical difference (p<0.05). *Wilcoxon Rank-Sum test **Chi-Square test.

Multivariate analysis using the continuous model showed that districts with a higher percentage of the population living more than five km from a health facility [relative risk (RR): 1.025 per 1% increase; 95% CI: 1.010, 1.039] or predominantly practicing Islam [RR: 1.013 per 1% increase; 95% CI: 1.001, 1.025] had a higher risk of cholera. Districts that bordered a large body of water had a 5.5-fold risk of cholera compared to districts that did not border a large body of water [95% CI: 2.472, 12.404]. Further analysis separating districts bordering ocean from those bordering a fresh body of water revealed that the risk of cholera among districts bordering ocean [RR: 18.07, 95% CI: 1.17, 278.04] was not significantly different from the risk among those districts bordering fresh water [RR: 19.91, 95% CI: 2.34, 169.14]. Increased rainfall from October to December was associated with an increased risk of cholera [RR: 1.003 per 1 mm increase; 95% CI: 1.001, 1.005]. Increased rainfall from April to June was associated with a decreased risk of cholera [RR: 0.996 per 1 mm increase; 95% CI: 0.992, 0.999]. There was no detectable association between cholera incidence and population density [RR: 1.000 per 1 mm increase; 95% CI: 0.9996, 1.0002], poverty levels [RR: 1.006; 95% CI: 0.986, 1.026], piped water [RR: 0.993; 95% CI: 0.974 1.012], sanitary facilities [RR: 1.003; 95%CI: 0.988, 1.018], rainfall from January to March [RR: 1.000; 95% CI: 0.997, 1.004], or rainfall from July to September [RR: 1.002; 95% CI: 0.999, 1.005]. The covariate quantifying the percentage of the population with no schooling was removed from the multivariate analysis due to a variance inflation factor above five.

As a sensitivity analysis, we replaced all continuous covariates of interest with corresponding categorical variables. In this multivariate analysis, bordering a large body of water, lack of proximity to health facilities, lack of piped water, increased rainfall from October to December, and decreased rainfall from April to June were significantly associated with an increased risk of cholera (see Table 2).

**Table 2:** **District characteristics associated with adjusted risk of cholera in Kenya**

	RR (95% CI) categorical model	RR (95% CI) continuous model
*Population density*		0.9999 (0.9996, 1.0002)
0–50	Ref	
51–200	0.23 (0.05, 1.02)	
201–500	0.12 (0.02, 0.73)	
501–1000	0.17 (0.00, 9.88)	
1000+	0.77 (0.05, 10.83)	
*Poverty,%*		1.0059 (0.9864, 1.0257)
0–33%	Ref	
34–66%	6.01 (0.53, 67.70)	
67–100%	4.00 (0.38, 41.50)	
*Unsafe sanitary facilities,%*		1.0027 (0.9875, 1.0181)
0–20%	Ref	
21–40%	1.47 (0.2 7, 7.84)	
41–60%	1.44 (0.33, 6.20)	
61–100%	0.15 (0.01, 1.80)	
*No piped water,%*		0.9929 (0.9740, 1.0122)
0–50%	Ref	
51–75%	**5.21 (1.63, 16.64)**	
76–100%	**3.31 (0.97, 11.30)**	
*No health facility within 5 km,%*		**1.0245 (1.0101, 1.0390)**
0–25%	Ref	
26–50%	0.87 (0.06, 11.98)	
51–75%	1.64 (0.19, 13.72)	
76–100%	**10.80 (1.63, 71.57)**	
*Muslim,%*		**1.0130 (1.0011, 1.0250)**
0%	Ref	
1–10%	0.47 (0.05, 4.28)	
11–20%	1.67 (0.19, 14.27)	
21–100%	1.38 (0.29, 6.45)	
*Large body of water*		**5.5 (2.4724, 12.4044)**
No	Ref	
Ocean	**18.07 (1.17, 278.04)**	
Fresh	**19.91 (2.34, 169.14)**	
*Rain from January to March (mm)*		1.0002 (0.9966, 1.0038)
0–100 mm	Ref	
101–200 mm	1.85 (0.42, 8.16)	
201–299 mm	1.85 (0.59, 5.81)	
300+ mm	1.86 (0.52, 6.63)	
*Rain from April to June (mm)*		**0.9957 (0.9923, 0.9991)**
0–100 mm	Ref	
101–200 mm	**0.09 (0.01, 0.51)**	
201–299 mm	**0.15 (0.03, 0.59)**	
300+ mm	**0.03 (0.00, 0.18)**	
*Rain from July to September (mm)*		1.0020 (0.9991, 1.0049)
0–100 mm	Ref	
101–200 mm	1.10 (0.33, 3.59)	
201–299 mm	1.47 (0.24, 9.05)	
300+ mm	5.24 (0.60, 45.55)	
*Rain from October to December (mm)*		**1.0033 (1.0013, 1.0053)**
0–100 mm	Ref	
101–200 mm	6.56 (0.54, 79.79)	
201–299 mm	**17.98 (1.54, 209.70)**	
300+ mm	**24.52 (2.21, 271.90)**	

Bolded values represents significant statistical difference (p<0.05).

## Discussion

Our study provides strong evidence for an association between rainfall and reported cholera cases. Interestingly, the direction of these associations was subject to seasonal variability. From April to June, districts with increased rainfall had a decreased risk of cholera, while districts with increased rainfall between October and December had an increased risk of cholera. These two time periods correspond to the two rainy seasons in Kenya: the long rainy season from late March to May and the short rainy season from October to December. These results are consistent with prior studies, which have demonstrated both an increased risk [8, 11–13, 26–28] and a decreased risk of cholera outbreaks [29] with increases in rainfall during the various rainy seasons. We hypothesize that a lack of rainfall during the long rainy season may insufficiently decrease the salinity of the water supply, providing optimal conditions for the copepod population to flourish and facilitate an increase in cholera cases. Conversely, an abundance of rainfall during the short rainy season may result in extended floods, which concentrate the population in drier areas and break down sanitary conditions, promoting cholera transmission through the more direct fecal-oral route. However, the ecologic nature of this study prevented us from further investigating physico-chemical and biological properties of water bodies associated with a higher risk of cholera. Further studies, therefore, should include these properties.

In our analysis, the results suggest that districts with a higher percentage of residents who practice Islam have an increased risk of cholera in Kenya, which may result from the use of a communal bucket to wash hands before entering a mosque and eating communal food. This phenomenon is similar to the findings of Mugoya et al. [30] who showed that funeral attendance is a risk factor for cholera in Africa, possibly from eating communal food. However, this finding may be a marker for an alternative, underlying factor.

Our study showed that a lack of piped water is associated with an increased risk of cholera outbreaks in Kenya, which is in accordance with several other studies [24, 31]. Considering the success of population-level interventions such as increased potable water in reducing diarrhea-related mortality in developing countries [31], major nationwide investments in drinking water and sanitation infrastructure are recommended to reduce the incidence of cholera in Kenya.

To our knowledge, this is the first study that has made an association between distance to health facility and cholera outbreaks. However, other studies have hypothesized that the persistently high case-fatality rate of cholera in Sub-Saharan Africa may reflect more general problems of access to effective health care, including access to lifesaving rehydration therapy [5, 31].

Eighteen out of the 69 districts (26.1%) in Kenya share a border with three large bodies of water: the Indian Ocean, Lake Victoria, and Lake Turkana. Our study showed that these districts had an increased risk of cholera, which reinforces previous studies showing that drinking water from Lake Victoria was a risk factor for cholera [15, 16] and was associated with the lake’s yearly water hyacinth coverage [15]. Several previous studies also observed a significant link between cholera outbreaks and communities’ locations being close to body of waters [8, 11–13]. We did not, however, observe any difference between communities bordering lakes or ocean. Besides using fresh water as a water resource, fishing in coastal communities, sea water temperature, and plankton activity [8, 11, 12] might explain this observation. This study failed to detect statistically significant associations for many characteristics previously shown to be risk factors for cholera, including population density [17–22], poverty [20–22], and unsafe sanitary facilities [23, 24]. We note that there was not much difference in the relative frequency of these characteristics across the 69 districts in Kenya. This lack of variability, together with a lack of statistical power, may explain why we failed to detect significant associations between these characteristics and cholera at district level.

We recognize several limitations of our study. The quality of the secondary data used for the analysis may be limited. For example, cholera surveillance and reporting rates may not be consistent among districts, particularly in rural areas where access to health care can be difficult. In addition, the number of cholera cases for 1999 and 2000 are approximate figures reported in intervals of 50. Second, data regarding many risk factors previously linked to cholera outbreaks—cultural practices such as eating communal food at funerals, hygienic practices such as washing hands without soap, drainage networks, yearly water hyacinth coverage, and other climatic factors like water temperature—were not available at district level in Kenya and thus could not be investigated. Lastly, the spatial scale of the data defined by administrative boundaries was generally large, limiting the spatial patterns that can be detected. In addition, the high-risk area for cholera outbreaks cannot determine the actual risk within a smaller group of people within a district. Therefore, applying the results at a location level may be difficult.

## Conclusion

This study demonstrated that bordering a large body of water, lack of nearby health facilities, increased rainfall from October to December, and decreased rainfall from April to June are all significantly associated with an increased risk of cholera in Kenya. It is therefore necessary for health officials and policymakers to identify these high-risk areas and make appropriate health planning and resource allocations to decrease the risk of cholera. This study also highlights the importance of adequate surveillance systems to monitor disease occurrence.

## Electronic supplementary material

Additional file 1: **Multilingual abstracts in the six official working languages of the United Nations.** (PDF 309 KB)

## Abbreviations

DDSR: Division of Disease Surveillance and Response
ESRC: Ethical and Scientific Review Committee
FAO: Food and Agriculture Organization
GIS: Geographic Information System
IRB: Institutional Review Board
RR: Relative risk
VIFs: Variance inflation factors.
