# Supplementary material for: Interaction between climatic, environmental, and demographic factors on cholera outbreaks in Kenya
Source: Infect Dis Poverty. 2014 Oct 1;3:37. doi: 10.1186/2049-9957-3-37 (PMC4200235; doi:10.1186/2049-9957-3-37)
Supplement: Supplementary file 1 — Additional file 1: Multilingual abstracts in the six official working languages of the United Nations. (PDF 309 KB) [file 40249_2014_79_MOESM1_ESM.pdf]

Translation of the abstract into the six official working languages of the United Nations

التفاعل بين العوامل المناخية والبيئية والديموغرافية في تفشي مرض الكوليرا في كينيا

جيمس د. ستولتسفاس، جين كارتر، ميوج أكبينار-إلسي، مارتن ماتو، فيكتوريا اكيموتو، مارك جيجانتي، دانيال لانجات، أمور سينار إلسي

ملخص

خلفية: يظل مرض الكوليرا مصدر قلق هام للصحة العامة في البلدان النامية بما في ذلك كينيا، حيث سجلت 11،769 حالة مرضية و274 حالة وفاة في عام 2009 وفقًا لمنظمة الصحة العالمية (WHO). هذه الدراسة البيئية تدرس تأثير المتغيرات المختلفة المناخية والبيئية والديموغرافية على التوزيع المكاني لحالات الكوليرا في كينيا.

الطريقة: تم جمع البيانات على مستوى المقاطعات من قسم كينيا لمراقبة الأمراض والاستجابة ومن قسم الأرصاد الجوية ومن المكتب الوطني للإحصاء. وتضمنت البيانات التعداد السكاني لكينيا من 1999 حتى 2009.

النتائج: أظهرت التحاليل متعددة المتغيرات أن خطر تفشي مرض الكوليرا في المقاطعات تزايد كلما زادت نسبة السكان الذين يعيشون بعيدًا عن المرافق الصحية بمسافة أكثر من خمسة كيلومترات (اختطار نسبي RR: 1.025 لزيادة بنسبة 1% - 95% حدود الثقة CI: 1.1010، 1.039) – السكان الذين يحدهم جسم مائي (اختطار نسبي RR: 5.5 - حدود الثقة CI 95%: 2.472، 12.404) – يشهدون زيادة في هطول الأمطار من أكتوبر – تشرين الأول حتى ديسمبر – كانون الأول (اختطار نسبي RR: 1.003 لكل 1ميليمتر زيادة: 95% حدود الثقة CI: 1.001، 1.005) – يشهدون نقصًا في هطول الأمطار (اختطار نسبي RR: 0.996 لكل 1ميليمتر زيادة: 95% حدود الثقة CI: 0.992، 0.999).

لم يكن هناك ارتباط واضح بين مرض الكوليرا والكثافة السكانية أوالفقر أوتوفر مياه الأنابيب أوطرق التخلص من النفايات أوهطول الأمطار من يناير- كانون الثاني حتى مارس- آذار أو هطول الأمطار من يوليو- تموز حتى سبتمبر-أيلول.

استنتاج: العيش على حدود جسم مائي ونقص المرافق الصحية القريبة وتغيرات هطول الأمطار، كلها من العوامل المرتبطة بصورة كبيرة مع زيادة خطر انتشار مرض الكوليرا في كينيا.

Translated from English version into Arabic by T. Catherine Hanna, through

TRANSLATORS
WITHOUT BORDERS

气候环境因素和人口学因素对肯尼亚霍乱暴发的交互影响分析

James D. Stoltzfus, Jane Y. Carter, Muge Akpinar-Elci, Martin Matu, Victoria Kimotho, Mark J. Giganti, Daniel Langat, Omur Cinar Elci

摘要

引言：霍乱仍然是发展中国家重要的公共卫生问题。根据世界卫生组织报告显示，2009 年肯尼亚有 11,769 例霍乱病例报告，其中 274 例死亡。本研究采用生态学研究方法，调查不同气象因素、环境因素以及人口学因素对霍乱病例空间分布的影响。

方法：以地区为单位收集数据。数据来源为肯尼亚疾病监测与反应办公室，气象部门以及国家统计局。数据包括 1999 年至 2009 年的人口总数。

结果：多元统计分析结果显示，在一个地区导致霍乱暴发风险增加的因素有：居住在距离医疗机构 5 公里以外（每增加 1% 的 RR: 1.025；95% CI: 1.010, 1.039），临近水体居住(RR: 5.5; 95% CI: 2.472, 12.404)，10 月至 12 月降雨增多(降雨量每增加 1 毫米 RR: 1.003; 95% CI: 1.001, 1.005)，和 4 月至 6 月降雨量减少(降雨量每减少 1 毫米 RR: 0.996; 95% CI: 0.992, 0.999)。而霍乱的发生与人口密度、贫困程度，管道水的可及性，废水处理方式以及 1 月至 3 月和 7 月至 9 月的降雨量等无关。

结论：居住在大型水体旁，周围缺少医疗机构以及降雨量的变化等因素与肯尼亚霍乱发病风险增加显著相关。

Translated from English version into Chinese by Zhang Shao-sen, through

CDC CHINESE CENTER FOR DISEASE CONTROL AND PREVENTION
NATIONAL INSTITUTE OF PARASITIC DISEASES

Interaction entre les facteurs climatiques, environnementaux et démographiques sur les épidémies de choléra au Kenya.

James D. Stoltzfus, Jane Y. Carter, Muge Akpinar-Elci, Martin Matu, Victoria Kimotho, Mark J. Giganti, Daniel Langat, Omur Cinar Elci

Description

Contexte : Le choléra reste un problème de santé publique majeur dans les pays en voie de développement notamment au Kenya où 11 769 cas et 274 décès ont été recensés en 2009 par l'Organisation Mondiale de la Santé (OMS). Cette étude écologique met en avant l'impact de divers facteurs climatiques, environnementales et démographiques sur la répartition géographique des cas de choléra au Kenya.

Méthodes : Les données de chaque région ont été recueillies auprès de la Division de Surveillance des Maladies et d'Intervention du Kenya, du Département Météorologique et du Bureau National des Statistiques. Les données incluent toute la population kenyane entre 1999 et 2009.

Résultats : Les analyses à plusieurs variables ont montrées qu'il y avait plus de risques d'épidémie de choléra dans les régions où une plus grande partie de la population vivait à plus de cinq kilomètres d'un établissement de santé (RR : 1,025 pour 1% d'augmentation ; 95%, IC : 1,010, 1,039), les régions à proximité d'une étendue d'eau (RR: 5,5 ; 95%, IC : 2,472, 12,404), les régions touchées par de fortes pluies entre octobre et décembre (RR : 1,003 pour 1% d'augmentation ; 95%, IC : 1,001, 1,005) et les régions qui ont connu une diminution des précipitations entre avril et juin (RR: 0,996 pour 1% d'augmentation ; 95%, IC : 0,992, 0,999). Aucun lien de causalité n'a été clairement établi entre l'épidémie de choléra et la densité de population, la pauvreté, l'approvisionnement en eau courante, les méthodes d'élimination des déchets, les pluies entre janvier et mars et entre juillet et septembre.

Conclusion : Le proximité d'une étendue d'eau, l'absence d'établissement de santé de proximité et les modifications de la pluviométrie ont une incidence importante sur l'augmentation des risques de choléra au Kenya.

Translated from English version into French by Fanny, through

TRANSLATORS
WITHOUT BORDERS

Связь между факторами, определяющимися климатом, окружающей средой и демографией, применительно к вспышке холеры в Кении.

James D. Stoltzfus, Jane Y. Carter, Muge Akpinar-Elci, Martin Matu, Victoria Kimotho, Mark J. Giganti, Daniel Langat, Omur Cinar Elci

Отрывок

Предпосылки: холера остается важной проблемой здравоохранения в развивающихся странах, включая Кению, где в 2009 году, согласно ВОЗ, было зафиксировано 11 769 случаев холеры, включая 274 с смертельным исходом. Это экологическое исследование изучает влияние факторов окружающей среды, климата и демографии на рост заболеваемости холерой на территории Кении.

Методы: данные по районам сбирались с помощью Дивизиона борьбы с заболеваниями в Кении (Kenya's Division of Disease Surveillance and Response), Метеорологической службы, Национального бюро статистики. Данные представляют все население Кении с 1999 по 2009 год.

Результаты: вариативная обработка данных показала, риск вспышки холеры повышается в районах, где большая часть населения проживает более чем в 5 км от учреждения здравоохранения (RR: 1.025 увеличение на 1%; 95% CI: 1.010, 1.039), расположенных рядом с большим водоемом (RR: 5.5; 95% CI: 2.472, 12.404), имеющих увеличение объемов дождевых масс с апреля по июнь (RR: 0.996 на 1 мм увеличения; 95% CI: 0.992, 0.999). Не прослеживается четкая связь между холерой и плотностью населения, бедностью, доступностью водопроводной воды, методами утилизации отходов, объемом дождевых масс с января по март и с июля по сентябрь.

Вывод: на рост риска холеры в Кении имели значительное влияние такие факторы, как расположение рядом с большими водоемами, отсутствие здравоохранительных учреждений рядом поселениями, изменение объемов дождевых масс в регионах.

Translated from English version into Russian by Anna Philippova, through

TRANSLATORS
WITHOUT BORDERS

Relación entre factores climáticos, ambientales y demográficos con los brotes de cólera en Kenia

James D. Stoltzfus, Jane Y. Carter, Muge Akpinar-Elci, Martin Matu, Victoria Kimotho, Mark J. Giganti, Daniel Langat, Omur Cinar Elci

Resumen

Antecedentes: El cólera sigue siendo un gran problema de salud pública en los países en vías de desarrollo incluido Kenia, donde, según la Organización Mundial de la Salud (OMS), se declararon 11.769 casos y 274 fallecimientos en 2009. El presente estudio ecológico investiga el impacto de las distintas variables climáticas, ambientales y demográficas sobre la distribución espacial de los casos de cólera en Kenia.

Métodos: Los datos a nivel distrito fueron recabados por la División de Observación y Respuesta ante las enfermedades, el Departamento Meteorológico y la Oficina Nacional de Estadística de Kenia. Se incluyó en los datos a la población total de Kenia de 1999 a 2009.

Resultados: El análisis multivariante mostró que el riesgo de brotes de cólera se había incrementado en los distritos toda vez que una gran parte de la población residiera a más de cinco kilómetros de un centro sanitario (RR: 1,025 por cada aumento del 1%; IC del 95%: 1,010-1,039), a la orilla de una extensión acuática (RR: 5,5; IC del 95%: 2,472-12,404), sufriese un aumento de las lluvias de octubre a diciembre (RR: 1,003 por cada aumento de 1 mm; IC del 95%: 1,001-1,005), o una disminución de las lluvias de abril a junio (RR: 0,996 por cada aumento de 1 mm; IC del 95%: 0,992-0,999). No se detectó ninguna asociación entre cólera y densidad de población, pobreza, disponibilidad de agua corriente, sistema de eliminación de residuos, lluvias de enero a marzo o lluvias de julio a septiembre.

Conclusión: Vivir a la orilla de una gran extensión de agua, la carencia de centros sanitarios cercanos y los cambios en las lluvias estaban asociados de manera significativa con un aumento en el riesgo de cólera en Kenia.

Translated from English version into Spanish by Raquel Bentué, through

TRANSLATORS
WITHOUT BORDERS
